# Investigating Cumulative Exposures among 3- to 4-Year-Old Children Using Wearable Ultrafine Particle Sensors and Language Environment Devices: A Pilot and Feasibility Study

**DOI:** 10.3390/ijerph17145259

**Published:** 2020-07-21

**Authors:** Amy A. Schultz, Kristen M.C. Malecki, Maddie M. Olson, Saliha B. Selman, Oona-Ife Olaiya, Alexandra Spicer, James J. Schauer, Ross Edwards, Heather L. Kirkorian, Janean Dilworth-Bart

**Affiliations:** 1 Department of Population Health Sciences, School of Medicine & Public Health, University of Wisconsin—Madison, Madison, WI 53706, USA; aaschultz4@show.wisc.edu (A.A.S.); mmolson7@wisc.edu (M.M.O.); aspicer@medicine.wisc.edu (A.S.); 2 Molecular and Environmental Toxicology Program, School of Medicine & Public Health, University of Wisconsin—Madison, Madison, WI 53706, USA; 3 Department of Civil & Environmental Engineering, University of Wisconsin—Madison, Madison, WI 53706, USA; sselman@wisc.edu (S.B.S.); jjschauer@wisc.edu (J.J.S.); pedwards5@wisc.edu (R.E.); 4 Human Development and Family Studies Department, School of Human Ecology, University of Wisconsin—Madison, Madison, WI 53706, USA; oolaiya24@gmail.com (O.-I.O.); kirkorian@wisc.edu (H.L.K.); jedilworth@wisc.edu (J.D.-B.)

**Keywords:** PM2.5, air pollution, noise, child development, exposure assessment, ultrafine particles, cumulative exposure

## Abstract

Interdisciplinary approaches are needed to measure the additive or multiplicative impacts of chemical and non-chemical stressors on child development outcomes. The lack of interdisciplinary approaches to environmental health and child development has led to a gap in the development of effective intervention strategies. It is hypothesized that a broader systems approach can support more effective interventions over time. To achieve these goals, detailed study protocols are needed. Researchers in child development typically focus on psychosocial stressors. Less attention is paid to chemical and non-chemical stressors and how the interaction of these stressors may impact child development. This feasibility study aims to bridge the gap between child development and environmental epidemiology research by trialing novel methods of gathering ultrafine particle data with a wearable air sensor, while simultaneously gathering language and noise data with the Language Environment Analysis (LENA) system. Additionally, psychosocial data (e.g., parenting quality, caregiver depression, and household chaos) was gathered from parent reports. Child participants (age 3–4 years) completed cognitive tasks to assess self-regulation and receptive language skills, and provided a biospecimen analyzed for inflammatory biomarkers. Data collection was completed at two time points, roughly corresponding to fall and spring. Twenty-six participants were recruited for baseline data, and 11 participants completed a follow-up session. Preliminary results indicate that it is feasible to gather personal Particulate Matter (PM2.5), language, and noise data, cognitive assessments, and biospecimens from our sample of 3-4-year-old children. While there are obstacles to overcome when working with this age group, future studies can benefit from adapting lessons learned regarding recruitment strategies, study design, and protocol implementation.

## 1. Introduction

In an increasingly complex world, it is recognized that traditional environmental health research evaluating chemical and non-chemical stressors separately is insufficient in addressing future challenges, and more complex approaches and interdisciplinary strategies are needed [1,2,3,4,5,6]. Chemical and non-chemical stressors often co-occur, particularly in more economically vulnerable populations [7]. More sophisticated interdisciplinary approaches to measuring the additive or multiplicative impacts on child developmental outcomes are needed [8,9,10,11,12]. Environmental epidemiologists are often trained in exposure science, with less attention to understanding the subtle impacts on child development. Similarly, scholars in child development focus on psychosocial processes with less attention towards understanding how psychosocial and chemical factors may intersect to exacerbate or protect child growth and development. Furthermore, research has focused on environmental exposures during the pre- and early post-natal periods with less emphasis on exposures during early childhood, a time of rapid neurological and physical development [13,14,15]. Finally, children live in families and communities, and their interactions, resources, and assets likely also play a significant role in mitigating or exacerbating child outcomes [16,17]. Therefore, to truly capture the unique cumulative exposures to physical, social, and behavioral risk factors, multi-disciplinary teams, including both environmental and social scientists, are needed. 

Interdisciplinary teams can support measurement of unique features of a child’s microenvironment (e.g., noise and air pollution) as well as outcomes (e.g., learning and growth) and build a foundation for future innovative research. Existing studies within child development or environmental health disciplines either address issues of family and social context or environmental risk factors and child development outcomes. However, systems science would argue that risk factors contributing to child growth, support, and resilience are often inextricably linked. Further, many of these factors are often highly correlated. Further, addressing social factors without addressing the physical environment may limit opportunities to fully understand the interaction and mediation between risk factors. Effective interventions and prevention strategies often fall short, likely due to a lack of empirical data supporting these complex relationships. To date, few study protocols that bridge the unique aspects of environmental epidemiology, social psychology, and child development have been published. 

The Cumulative Risks, Early Development, and Emerging Academic Trajectories (CREATE) project aims to address these gaps. The goal of this first study is to determine the feasibility of examining the cumulative and interactive effects that psychosocial, non-chemical, and chemical stressors have on preschool-aged (three to four years) children’s academic readiness. The study was conducted between Fall 2017 and October 2019 by a collaborative team of investigators at the University of Wisconsin, Madison. Experts in child development and environmental health sciences (including epidemiology and environmental engineering) collaborated on the development of the underlying theory, protocols, and data collection methods to achieve study aims in this often hard-to-reach population of young children. CREATE capitalizes on recent technological advancements to refine measurement of real-time, personal exposure to environmental stressors and captures a multitude of psychosocial stressors in a child’s microenvironment over a one-year time period. The following describes the background and rationale, methods and materials, and feasibility for such an endeavor. The overall goals of this paper are to present the feasibility of collecting rigorous data regarding personal exposure in multiple settings and family and child development as well as unique information on social interactions through the measurement of child language data. A discussion of the key lessons learned is also provided. This paper serves as a reference for child development specialists interested in understanding physical environmental toxicant exposure measurement, and conversely provides details regarding child development measurements. A discussion of the key lessons learned is also provided as a guide to support future multidisciplinary child development and environment research.

**Figure 1:**
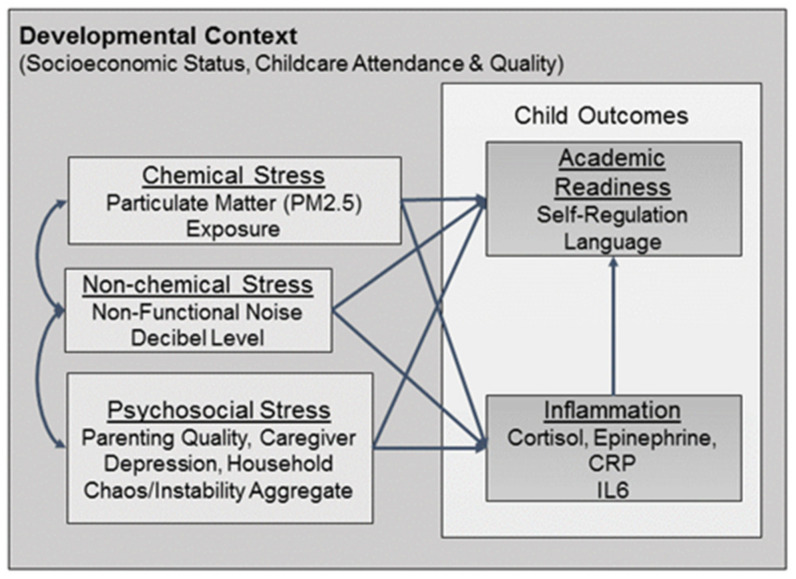
Schematic displaying the complex interactions between chemical, non-chemical, and psychosocial stressors, and child outcomes, including academic readiness and inflammation. PM2.5: Fine Particulate Matter <2.5 micrograms per cubic meter air (ug/m^3^); CRP: C-Reactive Protein; IL6: Interluekin-6.

CREATE is firmly grounded in developmental ecological perspectives [18], and posits that multiple risks have both direct effects on academic readiness and effects that are mediated by inflammation. The extent to which these stressors impact outcome is also dependent on child characteristics and interactions with salient individuals in the child’s daily life (Figure 1). Therefore, we took into consideration the major developmental tasks, contexts, and relationships of the early lifespan when developing the protocol. Of particular interest are the child cognitive and behavioral skills underlying academic readiness and achievement, the quality of the home and parenting environments, and socioeconomic status. We also attempted to assess the cumulative impacts of maternal psychological distress [18,19], familial socioeconomic status [18], parental warmth and consistency [20], and parental self-efficacy [21] given research indicating that deficits in these areas are significantly associated with lower child wellbeing, executive function, and academic performance [21].

Additionally, the protocol includes physical environmental stressors like noise and fine Particulate Matter (PM2.5) because they have also been shown to have similar negative impacts on children’s academic readiness as psychosocial stresses. For example, chronic exposure to noise from roadways or airports is associated with impairments in focused attention [3], literacy [1], and pre-literacy skills [2]. Ambient noise in the home has been shown to impact children’s time spent engaged in toy play and focused attention [3], the extent parents are engaged with and responsive to their children [4], and the quantity and quality of child-directed language in the presence of background television noise [4]. Similarly, fine particulate matter (PM2.5) in particular has been associated with deficits in child learning and working memory [5], cognitive development, including verbal intelligence, and visual motor abilities [19].

In this report we present results from the feasibility and acceptability of the CREATE protocol. This includes: (1) a description for methods used to simultaneously and longitudinally measure personal, real-time chemical (PM2.5), non-chemical (ambient noise), and psychosocial (caregiver depression, parenting quality, household chaos) stressors in children’s microenvironments (home, school, and ambient); and (2) preliminary findings regarding protocol adherence and feasibility.

## 2. Materials and Methods 

The CREATE feasibility study used a short-term longitudinal design to document the methods needed for the measurement and integration of multi-disciplinary data including psychosocial, chemical, and non-chemical stressors among three- to four-year-old children and their primary caregivers. The study was approved by the University of Wisconsin (2017-1174), Madison Institutional Review Board. Each wave of the feasibility study consisted of recruitment of preschool children and their primary caregiver to complete a three-day data collection protocol. This protocol was repeated at baseline (WAVE I) and follow-up (WAVE II). The assessments took place approximately six months apart, roughly corresponding to fall and spring. Longitudinal follow-up was conducted to assess changes in academic readiness over time. 

Baseline recruitment occurred at five sites. For the 2017–2018 school year, we enlisted three early childhood education centers in Madison, WI, two that served ethnically and socioeconomically diverse communities and one campus child development center that served predominantly white, higher Socio-Economic Status (SES) families. Initial recruitment protocols aimed to recruit several students from the same preschool; however, study personnel modified recruitment strategies and moved towards a referral-based recruitment strategy with much success [22]. We added a local community center and children’s museum as recruitment sites in 2018–2019. The schools, community center, and museum served as locations for study recruitment, consent, and data collection. Schools distributed recruitment flyers to parents of children aged three to four years. Study staff spent two to three days per week recruiting children and their primary caregivers at the entrance or exit of the schools during drop-off and pick-up hours.

Recruitment at the community center and the museum was less frequent and coordinated around events where three- to four-year-old children would be present with their parents. At recruitment, interested parties were screened for eligibility. If eligible, two home visits were scheduled on consecutive days, or contact information was received and home visits were scheduled later over the phone or by email.

**Figure 2:**
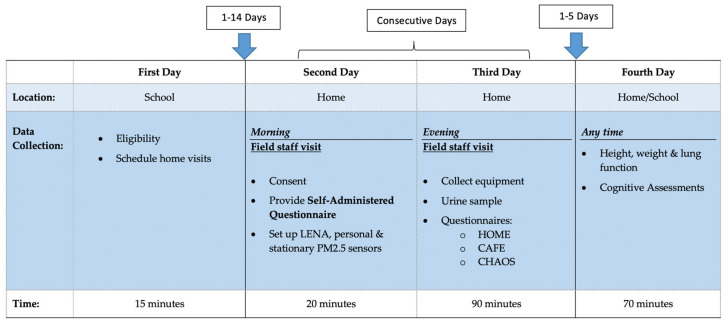
Description of the data collected for the feasibility study, organized by day of collection, location, and estimated time spent. Intervals between study days are labeled above the table. LENA: Language Environment Analysis System; HOME: Home Observation for Measurement of the Environment; CAFE: Comprehensive Assessment of Family Media Exposure; CHAOS: Confusion, Hubbub, and Order Scale.

Eligible child participants were between three and four years old, proficient in speaking and understanding English and ideally potty-trained. Children who were not potty-trained were still eligible for participation with the understanding that those children would likely opt out of urine sample collection. Caregivers had to be over the age of 18 years and proficient in understanding, reading, and speaking English. If more than one parent/guardian expressed interest in the study, only one self-selected to participate. Participants signed consent forms after staff determined them to be eligible. Participants were compensated with up to $200 for participating in all aspects of both baseline and follow-up visits. Meals were provided for families during the longer home visit (third day; Figure 2) to encourage participation and reduce participant burden.

The study protocol by day and data collection are summarized in Figure 2. Twenty six participants from wave I recruitment completed the baseline survey in the fall of 2017 or spring of 2018 and eleven were followed up 2–7 months after baseline (Wave II) during the summer or fall of 2019. The study is designed to have repeated protocols across time, allowing for both cross-sectional associations in cumulative exposures in association with baseline inflammatory and other biomarkers, but is also designed to track child-growth trajectories overtime. The latter allows for individual children to serve as their own control. Data can be analyzed using mixed effects models to account for variability across and within individuals and families. Both waves included the full three-day protocol including all home, personal and school environmental measurements, personal noise monitoring, and learning assessments.

**Figure 3:**
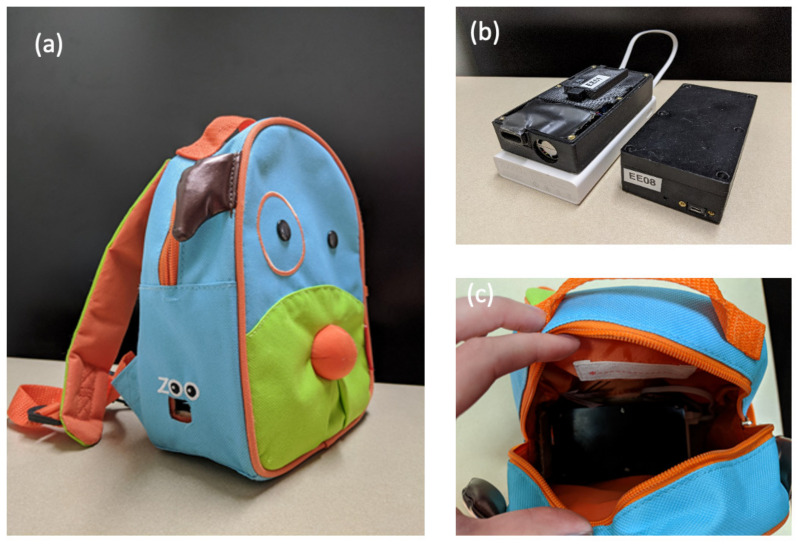
Photographs of the (**a**) exterior of backpack shown with inlet hole aligning with personal air sampler inlet; (**b**) PLANTOWER PM5003 air sampler with mobile battery pack (**left**) and with case securely fastened by screws (**right**) (**c**) PLANTOWER PM5003 personal air sampler and battery pack secured in child’s backpack.

The primary exposures of interest were air quality in the child’s microenvironments (home, commute, and school), non-functional noise, and parenting and home environment quality. Air quality was measured using two stationary and one personal monitor for each participant, which were specifically built for this project from PM 3003 particle sensors (Plantower CO, Ltd, Beijing, China). All three monitors were identical and collected ultra-fine and fine particulate matter (PM2.5 and PM10), carbon monoxide, temperature, and humidity data every minute. The air monitors were upgraded to PLANTOWER PM5003 sensors during the second year of the study to improve particle data quantity and reliability. The PM5003 sensor datasheet (Plantower CO, Ltd.) cites a maximum particle counting range from 0 to 1000 µg/m^3^, a maximum consistency error of ±10%, and a single response time of <1 s. The PM5003 sensors were operated in active mode with a data capture frequency of 1 s. The air monitors were contained inside plastic cases screwed closed, with a short air inlet and power cord holes. The plastic cases measured 7.5 × 7.5 × 5.5 inches (Figure 3a). 

To capture the child’s personal air exposure, backpacks were designed to securely hold a personal monitor and to be worn by the child for two consecutive days. The personal air monitor was powered by a portable battery pack (Zimi Corporation, PowerPack, 20,000 mAh) that lasted 55 h (Figure 3a). The weights of the battery pack, air sensor, and backpack are 8 ounces, 2 ounces, and 3 ounces, respectively, resulting in a total weight of 13 ounces. The personal air monitor was fixed with Velcro inside a child-sized backpack and zip-tied closed (Figure 3b). An air inlet was cut out of the backpack to allow unobstructed airflow into the air monitor (Figure 3c). The backpack was placed next to the child when sleeping and bathing, or when completing an activity where the backpack could not be worn due to discomfort.

To assess how well the personal monitors captured indoor environments, air monitors were set up in the participants’ pre-school classrooms at the start of recruitment and remained in the classrooms until participants had completed all aspects of the study. Data pertinent to individual participants were extracted and saved for dates of study participation. School air monitors were removed when the school was closed for summer break. Field staff checked on the air monitors periodically throughout the month to make sure they were still plugged in and collecting data. Data were extracted and downloaded off the school air monitors every two weeks.

At the first home visit, field staff also set up a stationary monitor in the participant’s home. The parent identified the room in the home where the child spends the most time (outside of the bedroom), and the monitor was placed in a location in that room where it could be plugged into an electrical outlet and unobstructed for two days. Parents were instructed to leave it alone, untouched, and plugged in until the following evening when field staff would collect the equipment.

Air monitors were taken out of the field every month and co-location quality control was run at headquarters. Home, school and personal monitors were run in conjunction with one another for 48 consecutive hours. Data were downloaded and to assess and compare different micro-environmental monitoring methods based on concordance with respect to daily average (mean, median), variation, and range. Data were also compared for quality control and monitors collecting inconsistent data compared to the fleet were re-calibrated before being re-entered into the field or were removed from the field altogether.

The child’s personal exposure to nonfunctional noise was measured using the Language Environment Analysis System (LENA Foundation, Boulder, CO, USA). The LENA system is a personal device that records 16 h of the child’s auditory environment. In addition to providing a decibel reader, it disentangles multiple sources of noise that can be considered functional (e.g., child-directed speech) or nonfunctional (e.g., traffic-related background noise). Data are collected every 5 min and an algorithm distinguishes sound segments as being (1) meaningful speech from the child or other nearby human or (2) non-meaningful noise such as television, background traffic, or construction noise. Meaningful speech is further processed into the number of conversational turns between the child and another person, the number of child vocalizations, and the number of adult words spoken. The non-meaningful noise is further identified: distant sound (from electronics such as television or radio), overlap (e.g., directed speech and electronic noise occurring at the same time), or silence (≤32 dB-SPL: decibels of sound pressure level).

**Figure 4:**
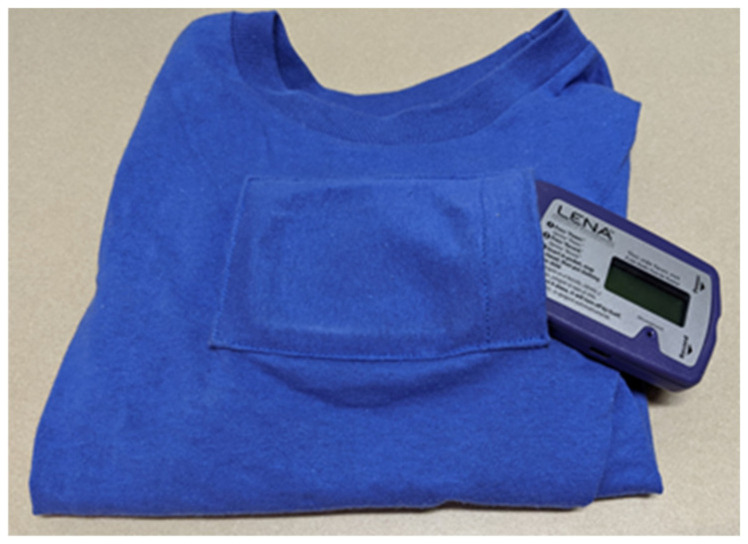
The Language Environment Analysis System (LENA Research Foundation, 2015) alongside the specially made t-shirt. The chest pocket secures the LENA throughout the day with a snap button.

The LENA device weighs 2 ounces and fits inside a pocket on the chest of a specially designed t-shirt (Figure 4). Children received two t-shirts on the morning of the first home visit, and they wore the shirts during the waking hours of two consecutive days. Parents and children were instructed and sent reminder texts or calls to turn the LENA off before bedtime and to turn the LENA on and wear it again once awake the next morning. Data were processed on-line via the LENA Research Foundation’s cloud-based processing system. Audio data were automatically and permanently deleted after processing, and there are no audio files or transcripts of the participants’ language environment.

Noise data were supplemented with a daily activity log from the Comprehensive Assessment of Family Media Exposure (café) Consortium assessment tools [23]. The diary was completed by the parent during the second home visit. The diary was presented through Qualtrics (Qualtrics, Provo, UT, USA) on a provided tablet. Parents were asked to walk through a typical 24-h day and recall typical activities and routines their child completes throughout the day, and whether screen media are being used during these activities. Primary activity categories included sleeping, eating, playing indoors, playing outdoors, using media, doing other household activities, traveling, and attending preschool or childcare.

Parenting quality, caregiver depression, and household chaos were collected from four different instruments and were aggregated to capture total psychosocial stress. The Home Observation for Measurement of the Environment (HOME) Inventory, Early Childhood Version [15] is a widely-used measure of home experience that includes observational and interview techniques to assess eight domains: learning materials, academic stimulation, parent modeling, language stimulation, variety, physical environment, parent responsiveness, and acceptance. Caregiver Depression was assessed with the Center for Epidemiological Studies Depression (CES-D) Scale, a 20-item measure of current depressive symptomatology [21,24]. Household chaos was assessed using the Confusion, Hubbub, and Order Scale (CHAOS), a 15-item parent-report measure of social-environmental confusion [7]. The Physical Health Questionnaire (PHQ-2), along with the Center for Epidemiological Studies Depression (CES-D) screener, were employed to assess the adult caregiver’s mental health. Primary outcomes included child verbal ability and self-regulation as measures of early child development as well as physical health and inflammatory markers collected via biospecimens.

On the last day of the three-day data collection protocol, children completed assessments of receptive vocabulary and self-regulation with two field staff members. The cognitive tasks assess different domains of academic readiness and were administered in a standardized order. Children followed along on a game board (see Supplementary Materials, Figure S1), which visualized progress toward completion of the cognitive assessments. Children received a book upon completion or attempted completion of the cognitive assessments. The child participant received a book after completing the cognitive assessments and could keep the backpack used for personal air monitoring after completing the follow-up visit. The Peabody Picture Vocabulary Test, IV (PPVT-IV) is a standardized assessment appropriate for use with individuals aged 2 years, 6 months to 90 years [25]. This measure assesses receptive vocabulary, i.e., the words an individual can comprehend. Self-regulation was assessed using multiple measures that will be composited for analysis. Head-Toes-Knees-Shoulders (HTKS) is a global assessment of self-regulation, the ability to control thoughts, behaviors, and emotions to achieve a goal [26,27]. The standard and advanced, “border” versions of the Dimensional Change Card Sort (DCCS) was used to assess the ability to shift attention [28]. Verbal Working Memory (VWM) and Nonverbal Working Memory (NVWM) were assessed using the standardized and normed verbal (VWM) and nonverbal (NVWM) working memory subtests, respectively, of the Stanford–Binet Intelligence Scales, 5th Edition [29].

Inflammatory markers were collected through urine sampling. Samples were collected from child participants during the second home visit. Parents were sent a reminder in the morning that the urine collection would occur in the late afternoon/evening visit. Soon after field staff arrived at the participant’s home, they placed a specimen collector on the toilet most frequently used by the child. The child participant was encouraged to attempt urine collection immediately. If urination was not immediately possible, the urine hat was left on the toilet for the child to use at any time during the 2-h visit. After the child participant urinated in the hat, field staff transferred urine into a sterile 60 mm plastic specimen cup, sealed it in a plastic bag, and placed it in a cooler with a cooler pack for the duration of the home visit and transport back to headquarters. Urine samples were immediately stored in a −80 °C freezer at headquarters before batch transport to the lab for analysis. Children received stickers after completing each biosample collection. After completion of all baseline and follow-up visits, urine samples were transported in a cooler of dry ice to the University of Wisconsin–Madison Primate Center Laboratories and analyzed for the following inflammatory markers: creatinine (adjustment factor), cortisol, interleukin 6, norepinephrine, and epinephrine.

Field staff measured the child’s weight (in kilograms) and height (in centimeters) using a portable stadiometer and scale during the last day of the three-day data collection protocol. Participants did not wear shoes for either measurement. The average height was taken from three consecutive measurements. Body Mass Index (BMI) was then calculated from the measured weight and height as kg/m^2^.

Forced Expiratory Volume in one second (FEV1) and Forced Vital Capacity (FVC) were measured via spirometry using an electronic peak flow meter (Jaeger AM, Yorba Linda, CA, USA), and validated protocol [30]. A small, disposable mouthpiece adapter designed specifically for young children was used (MicroGard II, Vyaire Medical, Inc. Mettawa, IL, USA) to assist children when using the device. Trained field staff gave study participants explicit directions on how to breathe into the spirometry device. Measurements were considered valid if two FEV1 and FVC readings were within 10% of the highest value measured. FEV1 to FVC ratio and percent predicted FEV1 (FEV1 divided by predicted FEV1) were also assessed to account for inter-individual variability in lung function measurement. Predicted FEV1 was calculated using sex, race, age, and height as defined by the National Health and Nutrition Exam Survey (NHANES) for the general US population [31].

After collecting wearables on the evening of day 2 of the protocol, the child was interviewed about the wearable devices, the backpack with the air sensor, and the special t-shirt with the LENA noise/language recorder (see Supplementary Materials, Figure S2). The child was asked whether the devices were annoying, painful, or uncomfortable to wear. They were also asked if they took the devices off at all and for long or short durations. The adult caregiver was given a short, self-administered survey regarding the wearable devices (see Supplementary Materials, Figure S2). Survey items asked whether the child complained about the devices, whether the child informed them that they took the devices off at school, or if the parent observed the child taking the devices off at home. The survey also inquired about whether adults would be interested in receiving noise or air data captured from the monitors.

## 3. Results

### 3.1. Study Sample

The characteristics of both the child and caregiver study participants are summarized in Table 1. Demographics were organized by recruitment method, where tabling events included recruitment at the preschools, museum, or community center, and referral-based included those who were referred by other participants or word of mouth. A total of 26 participants completed this study. A majority of participants were recruited from tabling events (n = 21 people) and a select few were recruited from referrals (n = 5 people). The average age of the children was 4.08 years, with the youngest participant being 3.08 years old and the oldest being 5.08 years old (at follow-up).

### 3.2. Data Completion

Data completion is summarized in Table 2. Among the 26 child participants, 21 (81%) and 25 (96%) participants completed the first day of baseline data collection of PM2.5 and LENA data, respectively. Data captured from the wearables dropped to 69% and 88% on the second consecutive day for PM2.5 and LENA at baseline, respectively. Only one participant was unable to complete the cognitive assessments, and only four of the 26 participants were unable to provide a urine sample. Of the 11 children who completed follow-up visits, PM2.5 data captured from the wearable device were collected on eight participants (73%) for both days of follow-up. Data from the LENA noise/language wearable increased to greater than 90% for both days of the follow-up. Cognitive assessments and urine samples were completed on all 11 children for follow-up. Lung function was captured on about 80% of the children for both baseline and follow-up. 

Data collected from the caregivers, which included the self-administered health survey as well as interviews, surveys, and observational assessments while in the home, were completed for 100% of caregivers. PM2.5 collections from stationary monitors in the home were captured successfully in 25 of the 26 households at baseline but dropped to 91% and 82% for day 1 and day 2 of home-based PM2.5 collection at follow-up, respectively. PM2.5 collections from the school sensors were poor, with 58% and 46% for day 1 and day 2 collection at baseline, respectively, and 27% and 18% for day 1 and day 2 at the follow-up visit, respectively. 

### 3.3. Participant Compliance 

Compliance of the wearable equipment was captured via an interview with parent and/or child after data collection (Table 3). Child participants were interviewed by field staff, and results were recorded if the field staff felt that the child provided a reliable, realistic report. Caregiver participants were provided with a survey with similar questions and additional questions regarding interest in receiving results from the feasibility study. Table 3 shows that children found backpacks to be annoying and a bit uncomfortable. Future studies should consider use of smaller, newer chip-based sensors that would minimize the need for large child backpacks. Similarly, recording the noise measurements was challenging, and over 40% of children found the device to be annoying and uncomfortable. At the same time, parents were very interested in receiving data from the study. Given the young age of the children in this study, the results are not surprising. At the same time, the findings point to the importance of considering the simplicity of measurement tools for real-time monitoring of young children.

### 3.4. PM2.5 and LENA Measurement

**Figure 5:**
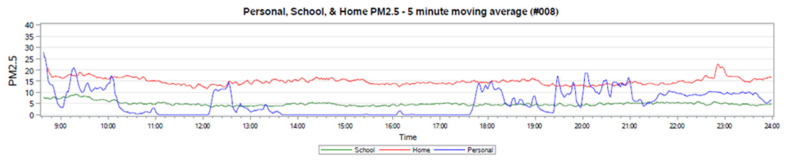
Time-series plot of PM2.5 data, averaged using a 5-min moving average. Data are included for air sensors from personal, home, and school monitors for participant #008.

Environmental data, including PM2.5, overall noise, and language were found to be feasible in this young age group of active 3–4-year-old children. Data were analyzed and presented here in time-series plots to demonstrate how PM2.5, noise, and language data fluctuate throughout the day. Personal, school, and home air sensors were plotted simultaneously for each participant (Figure 5). Data were collected each minute, and a 5-min moving average was calculated to smooth the time-series data. Descriptive statistics corresponding to the time-series plot in Figure 5 can be found in Table 4. Home and school monitoring data suggest relatively constant levels of air pollution exposure. By contrast, personal monitoring data for the children show large fluctuations throughout the day. From these data, the study investigators conclude that investments in personal monitoring are more important to capture child exposures than individual home or school measurements. Table 4 shows that the average values in both the school and home environment are captured by the personal monitoring, but the personal monitoring captures more variation throughout the day. Should peak exposures be important in child development, these would not be captured with either the school or the home environment monitoring. 

**Figure 6:**
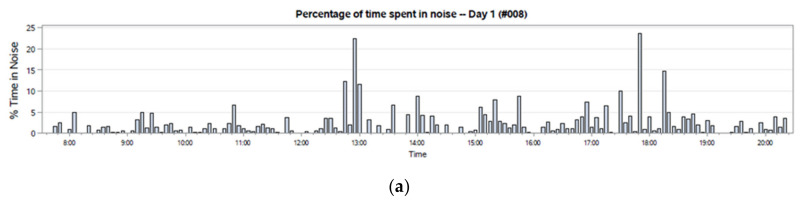

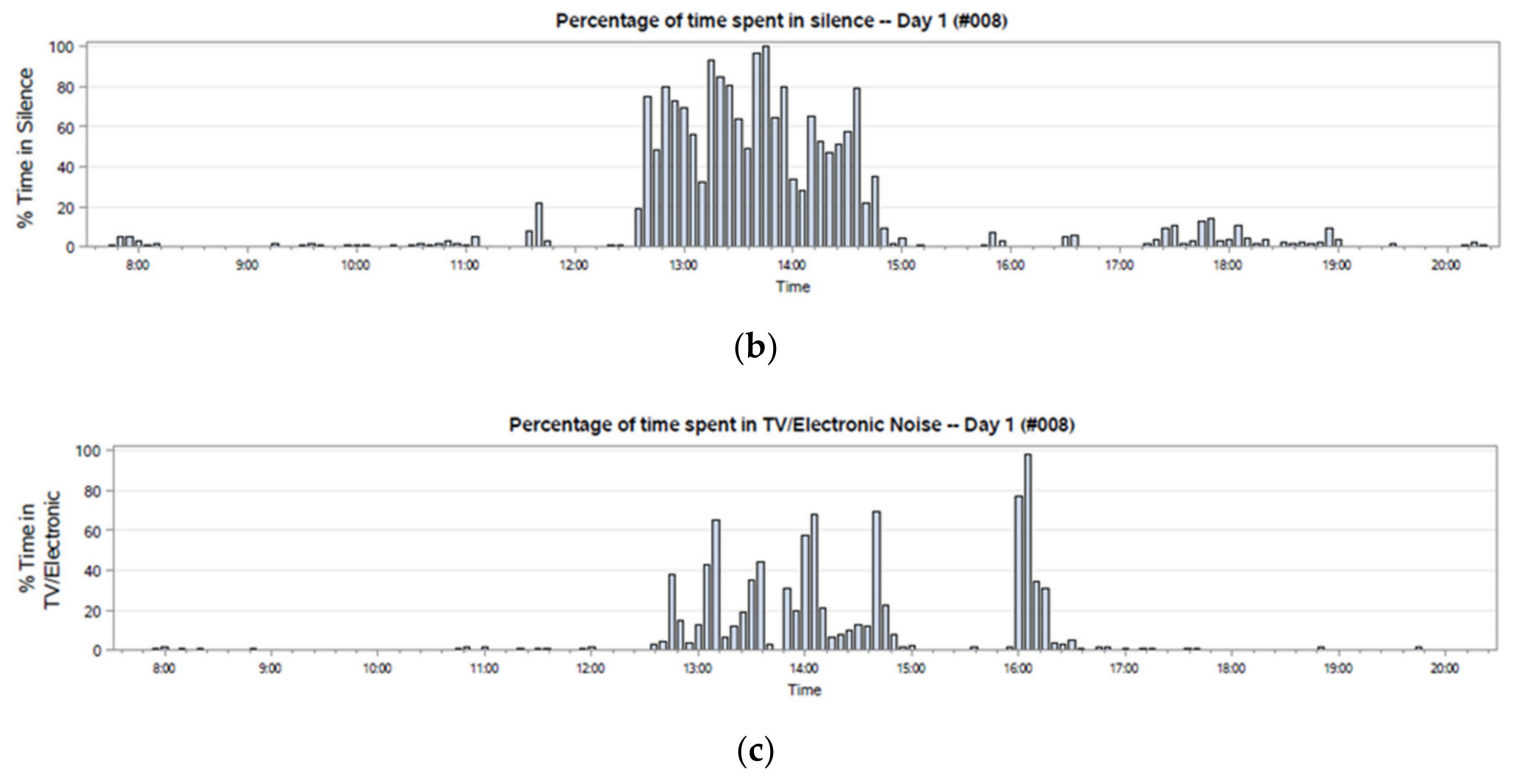
Bar graph of LENA data, specifically the percentage of time the recording was primarily noise (**a**), silence (**b**), and TV/Electronic noise (**c**) averaged across 5 min. Data are included for the day 1 recording for participant #008.

Similar time-series trends in language and noise exposure were monitored. A unique aspect of the LENA noise and language variables was also plotted in 5-min increments (Figure 6). However, LENA data are not always represented each minute, as the algorithm will analyze each minute of the recording and only quantify each variable if other variables are not present. For example, during a 1-min recording, the percent of time spent in noise will only be quantified in the absence of a meaningful variable, including adult word counts, conversational turns, and child vocalizations, and in the absence of electronic noise, silence, and overlap. Because this does not necessarily indicate an absence of noise in the 5-min interval, data were not visualized with a true “0”, as would happen in a time-series. A bar graph was chosen to represent these data, with the understanding that this would more accurately represent the data from moment to moment.

## 4. Discussion

The CREATE Study protocol supports the notion that we can now capitalize on the use of wearable technology to support more integrated protocols for the collection of real-time, personal noise and PM2.5 exposure among preschool children aged 3–4 years old [32]. The study also identifies the importance of engaging a multi-disciplinary team in the study design. Finally, the study offered important opportunities to identify limitations that can be used to refine protocols in the future. Capturing the multi-level components of social and environmental threats to child development during early childhood is unique and challenging. Most protocols to date have relied on testing of wearable air and noise sensors on older children, adolescents, and adults [32,33]. Few if any studies to our knowledge have tested the feasibility of using wearable technology to assess cumulative exposures in preschool children aged 3 to 4 years old [32,34]. 

The CREATE study adds to the growing evidence that suggests refined measurements of a child’s microenvironment are important, yet few studies have developed reliable protocols in this unique preschool-age population [11,35]. PM2.5 collections from individual participants demonstrate the value of collecting personal exposures from the wearable air sensors compared to stationary home and school environments. Results suggest investments should be made in developing devices that children can wear without much disruption. The PM2.5 personal monitors were worn in backpacks, which was found to be feasible, but almost one half of the participants found the backpacks to be annoying. Newer, smaller devices that are less bulky may improve tolerance and data collection. 

Protocols of this nature also requiring micro-environment exposure assessment and child observation require significant participant burden; thus careful attention must be paid to recruitment approaches. Initial recruitment protocols aimed to recruit several students from the same preschool; however, study personnel modified recruitment strategies and moved towards a referral-based recruitment strategy with much success [22]. Of all the recruitment locations (preschools, community center, and museum) and methods implemented, referrals resulted in the best retention, with 71% of those who initially expressed interest completing the study, compared to the 32% who were retained via tabling events. 

Recruitment from lower-income and racial/ethnic minority communities proved more challenging than recruitment in higher income, university-based preschools. Challenges in recruiting lower-income or minority participants are well established, including mistrust in research, lack of resources (e.g., reliable transportation, email, or phones), and risks associated with sensitive research questions [36]. Study teams worked in collaboration with preschool staff and families to overcome these barriers. Again, a combination of recruitment styles, and in particular referral-based sampling was most successful. Totals of 80% and 40% of participants from referral-based and tabling events, respectively, reported an annual individual income of less than $50,000. Although fewer participants were recruited with referral-based sampling, it is possible that had this method been implemented earlier, a more diverse demographic could have been recruited [37].

Future studies may benefit from using respondent-driven or snowball sampling among lower income populations and under-represented populations for which building trusted relationships remains a crucial component of research participation. Additionally, future studies would benefit from having established, mutually beneficial relationships with community partners and recruitment sites long before data collection begins [37]. Recruitment was most successful with the preschool whose relationship and ties with the field staff were strongest. Teachers and staff were more invested in ensuring classroom air monitors were left untouched and would advocate for and support the study among interested students and parents. This resulted in parents having greater trust and willingness to speak highly of and recommend the study to others. Field staff members engaged with the community beyond the study needs, and this was an important part of building a mutually beneficial relationship, enabling recruitment and participation in future studies.

Recruitment for research that assesses micro-environmental exposures and child observations requires significant participant burden. Therefore, careful attention to initial relationship building and recruitment is essential. Recruitment for CREATE was most successful when the project leaders and field staff created and maintained relationships with preschool recruitment sites. While recruitment itself was challenging overall, study completion was high for all components, with completion rates for most components ranging from 80%–100%. Several seemingly small but important factors were implemented that contributed to this study’s success and are worth mentioning as guides for future research in this area.

First, field staff had extensive experience and education working with children in research, clinical, and recreational settings, which better enabled them to connect with the children and families. Multiple encounters with field staff during recruitment at preschools helped facilitate building a trusting relationship, which may have helped children accept and comply with wearing the air and noise monitors. Two home visits with family were strategically placed prior to the last day of the data protocol where children are asked to complete physical measurements and cognitive assessments with field staff. These home visits enabled children to build a relationship with field staff and feel comfortable with them before their assessments. It was also held as a standard that the same two field staff members would see the family through the duration of the study. 

Second, children were provided with age-appropriate incentives. They were given different color choices for the special LENA t-shirt and were allowed to pick their favorite to wear. They were also given three different colorful animal backpacks to choose from (monkey, giraffe, and dog), which they would wear to house the air sensor. The freedom of choice among several fun and colorful options excited the children. Children were also given stickers for completing urine collection and a book for completing the cognitive assessments. After completion of follow-up, children were allowed to keep their backpack and a colorful bandage was placed over the air sensor inlet hole in the backpack. These small, child-friendly designs made the study engaging and exciting for child participants.

Third, our consistent and frequent quality control of the field equipment and data allowed us to make critical updates to the protocol as needed. For example, the PM3003 air sensors were conserving energy by collecting data at less frequent time intervals on the personal, battery-powered air sensor. This glitch was fixed by installing more battery packs. Frequent co-location quality control of air sensors enabled us to ensure all air data being collected were as accurate as possible. Feedback forms from participating children and adults resulted in the development of the cognitive assessments tracker board. Some children became restless to complete the series of cognitive assessments, but after creating a visual tracker for them to see their progress, child participants were more motivated to complete the assessments.

Despite these strengths, there were several challenges during the implementation that will inform the next stages of this research. While data completion was high for most study components, it was lower for day two of personal air quality data collection at baseline, and both days of personal air monitor data collection at follow-up; completion rates were intermittent for school air monitor data collection. The compliance survey indicated this was likely due to participant burden, as several reported the backpack was annoying or hurt to wear. Lighter and more compact batteries would help to alleviate the weight of the pack bothering the child participants. Since the bulkiness of the backpacks was also sometimes noted as uncomfortable during play, new designs and methods of portable devices for young children should continue to be investigated and developed. While missing personal air quality data were partly due to participant refusal or error, it was also due to the design of the air monitors. Occasionally, air monitors were returned to field staff unplugged yet still secured inside the backpack with no signs of tampering. Future personal, portable air sensors should be designed with a charging cord that cannot become loose. A charging cord secured into the air sensor with a locking feature would be best for children who run and play while wearing the air sensors. Moreover, future studies should include GPS trackers with the personal air sensor to accurately validate whether the data captured represented what the child was exposed to at all times. These data could be complemented with a daily log of the child’s activities throughout the day. Data completion was intermittent for school PM2.5 sensors. This was found to be attributed to a variety of factors, including non-attendance in school, sensors becoming unplugged at school and unnoticed until our weekly staff visits, and some referral-based participants not attending a preschool. 

## 5. Conclusions

Real-time data collection of air quality and noise using wearables on 3- to 4-year-old children presents many challenges, which are among the top reasons prior studies have not attempted to do so. For example, we found that children wearing backpacks during a 24-h period is tolerable for one day, but beyond that, many children are not comfortable or willing to wear backpacks. We also found that children may become overly curious and try to tamper with equipment. Moreover, urine sample collection among recently potty-trained (or in-training) children is a daunting task outside of a clinical setting. However, our feasibility study demonstrates these perceived barriers to data collection on 3- to 4-year-old children can be overcome. The success of this study can largely be attributed to the interdisciplinary team of experts from child development, environmental epidemiology, and environmental engineering that came together to develop and continually adapt a well thought-out protocol and study design. The feasibility of our study has important relevance for future research and intervention work. Future directions with the CREATE feasibility study will be to integrate key lessons learned from this feasibility study into a larger longitudinal design spanning several years throughout childhood and adolescence and will take lessons learned from this feasibility pilot in terms of recruitment strategies, study design, and protocol implementation.

## Figures and Tables

**Table 1:** Descriptive statistics for all participants, by recruitment method.

	Recruitment Method Referral Based Tabling Events	
Age of Child (in years)	% (n)	% (n)
3	80 (4)	50 (10)
4	20 (1)	50 (10)
Missing Gender of Child	N/A	1
Female	80 (4)	61.9 (13)
Male Child Race	20 (1)	38.1 (8)
White (non-Hispanic)	60 (3)	65 (13)
non-White	40 (2)	35 (7)
Missing Parent Education	N/A	1
Some college or less	0 (0)	4.76 (1)
At least a Bachelor’s degree	100 (4)	95.24 (20)
Missing Annual Household Income	1	N/A
<$50,000 / year	80 (4)	40 (8)
≥$50,000 / year	20 (1)	60 (12)
Missing Relationship Status	N/A	1
Married	100 (4)	85 (17)
Never Married	N/A	10 (2)
Living with Partner	N/A	5 (1)
Missing Household Members	1	1
2	N/A	14.29 (3)
3	N/A	33.33 (7)
4–5	75 (3)	47.62 (10)
6+	25 (1)	4.76 (1)
Missing	1	N/A

**Table 2:** Data completion table for all participants, by baseline and follow-up visits.

Data Collected	Baseline		Follow-Up	
	Attempted to Collect	n (%)	Attempted to Collect	n (%)
Personal PM2.5 Day 1 Personal PM2.5 Day 2	26 26	21 (81) 18 (69)	11 11	8 (73) 8 (73)
Home PM2.5 Day 1 Home PM2.5 Day 2	26 26	25 (96) 25 (96)	11 11	10 (91) 9 (82)
LENA Day 1 LENA Day 2	26 26	25 (96) 23 (88)	11 11	11 (100) 10 (91)
Health Questionnaire	26	26 (100)	11	11 (100)
CAF ^1^	26	26 (100)	11	11 (100)
Urine sample	26	22 (85)	11	11 (100)
Lung function via spirometry	26	20 (80)	11	9 (82)
Cognitive Assessments	26	25 (96)	11	11 (100)

^1^ Comprehensive Assessment of Family Media Exposure Consortium (CAFE).

**Table 3:** Data from the child and caregiver compliance interview and survey.

	Child Report		Caregiver Report	
	Baseline	Follow-Up	Baseline	Follow-Up
Backpack with personal air sensor				
Hurt to wear	8 (31%)	3 (27%)		
Annoying/Uncomfortable	14 (54%)	5 (45%)		
Child complained about backpack			9 (35%)	6 (55%)
T-shirt with noise & language monitor (LENA)				
Hurt to wear	7 (33%)	4 (40%)		
Annoying/Uncomfortable	10 (45%)	4 (44%)		
Child complained about t-shirt or monitor			12 (46%)	3 (27%)
Took t-shirt off	5 (24%)	2 (22%)	6 (24%)	2 (20%)
Removed noise monitor from t-shirt (at least once)	13 (62%)	4 (44%)		
Interest in receiving results				
Noise monitor (LENA) data			23 (92%)	9 (82%)
Air monitor data			24 (92%)	10 (90%)

**Table 4:** Descriptive statistics for participant #008. n is the number of observations, which were recorded for each minute. Min, max, mean, and median are all units of ug/m3.

	Home PM2.5	Personal PM2.5	School PM2.5
n	909	912	914
minimum	10	0	3
maximum	25	29	10
mean	14.92	5.47	4.91
median	15	4	5
standard dev.	1.78	5.63	1.13

## Supplementary Materials

The following are available online at https://www.mdpi.com/1660-4601/17/14/5259/s1, Figure S1: Visual schedule for cognitive assessments, Figure S2: Compliance interview and survey.
