# Supplementary material for: Investigating Cumulative Exposures among 3- to 4-Year-Old Children Using Wearable Ultrafine Particle Sensors and Language Environment Devices: A Pilot and Feasibility Study
Source: Int J Environ Res Public Health. 2020 Jul 21;17(14):5259. doi: 10.3390/ijerph17145259 (PMC7400160; doi:10.3390/ijerph17145259)
Supplement: Supplementary file 1 [file ijerph-17-05259-s001.zip › supplementary figure 2.pdf]

COMPLIANCE INTERVIEW

"Would it be okay if I asked you a few questions about the t-shirt and backpack you wore?"

If no, thank child for participating and move on to ask the parent/guardian questions.

- **Did wearing the backpack hurt?**
- Yes
- No
- Don't know
- Refused

- **Was wearing the backpack annoying or uncomfortable?**
- Yes
- No
- Don't know
- Refused

- **Did you take the backpack off during the day at all today?**
- Yes
- No
- Don't know
- Refused

- **How many times did you take the backpack off? _____________ Time(s)**
- **Did you leave it off for a long time (all school day) or short time?**
- Long time
- Short time
- Don't know
- Refused

- **Did you take the backpack off during the day at all yesterday?**
- Yes
- No
- Don't know
- Refused

- **How many times did you take the backpack off? _______________ Time(s)**
- **Did you leave it off for a long time (all school day, or all the time at home) or a short time?**
- Long time
- Short time
- Don't know
- Refused

- **Did the special t-shirt and noise monitor hurt to wear?**
- Yes
- No
- Don't know

- Refused

- **Was wearing the t-shirt and noise monitor annoying or uncomfortable?**
- Yes
- No
- Don’t know
- Refused

- **Did you take the t-shirt off during the day at all today?**
- Yes
- No
- Don’t know
- Refused

- **Did you take the noise monitor out of the t-shirt pocket today?**
- Yes
- No
- Don’t know
- Refused

  a. How many times did you take the t-shirt or noise monitor off today? _________ Time(s)

  b. Did you leave it off for a long time (all school day, or all the time at home) or a short time?
- Long time
- Short time
- Don’t know
- Refused

- **Did you take the t-shirt off during the day at all yesterday?**
  - Yes
  - No
  - Don’t know
  - Refused

- **Did you take the noise monitor out of the t-shirt pocket yesterday?**
  - Yes
  - No
  - Don’t know
  - Refused

- **How many times did you take the t-shirt or noise monitor off yesterday? _______ Time(s)**
- **Did you leave it off for a long time (all school day, or all the time at home) or a short time?**
  - Long time
  - Short time
  - Don’t know

- Refused

Date: ______________ **Study ID:** _______________

Compliance Survey

The following questions ask about your child's experience wearing the backpack and the special t-shirt, and your experience with the CREATE Project so far.

1. Did your child complain at all about wearing the backpack?

- ◯ Yes
- ◯ No
- ◯ Don't know

2. Did your child complain at all about wearing the t-shirt?

- ◯ Yes
- ◯ No
- ◯ Don't know

3. Did your child complain at all about wearing the noise monitor?

- ◯ Yes
- ◯ No
- ◯ Don't know

4. Did your child tell you that he or she took off the backpack at school?

- ◯ Yes
- ◯ No
- ◯ Don't know

5. Did your child tell you that he or she took off the t-shirt or noise monitor at school?

- ◯ Yes
- ◯ No
- ◯ Don't know

6. Did your child take off the backpack at all while at home (not incluing sleeping or bathing)?

- ○ Yes
- ○ No → **Go to question 9**
- ○ Don't know

7. About how many times did your child take off the backpack?

For example:

0	2

Time(s)

Fill in your answer below.

Time(s) ○ Don't Know

8. For about how long did your child take the backpack off each day?

For example:

0	1

Hour(s)

1	5

Minutes

Fill in your answer below.

Hour(s)

Minutes ○ Don't Know

9. Did your child take off the t-shirt or noise monitor while at home (not including sleeping or bathing)?

- ○ Yes
- ○ No → **Go to question 12**
- ○ Don't know

10. How many times per day did your child take off the t-shirt or noise monitor?

☐☐ Time(s)   ○ Don't Know

11. For how long did your child take the t-shirt or noise monitor off?

☐☐ Hour(s)   ☐☐ Minutes   ○ Don't Know

The following questions ask about your experience with the CREATE Project so far.

12. If given the option, would you like to receive results from the noise monitors?

- ○ Yes
- ○ No
- ○ Don't know

13. If given the option, would you like to receive results from the air monitors?

- ○ Yes
- ○ No
- ○ Don't know

14. Please provide any additional comments or feedback:

____

____

____

____
