# Supplementary figures and images for: Investigating Cumulative Exposures among 3- to 4-Year-Old Children Using Wearable Ultrafine Particle Sensors and Language Environment Devices: A Pilot and Feasibility Study
Source: Int J Environ Res Public Health. 2020 Jul 21;17(14):5259. doi: 10.3390/ijerph17145259 (PMC7400160; doi:10.3390/ijerph17145259)

# WHAT ARE WE DOING TODAY?

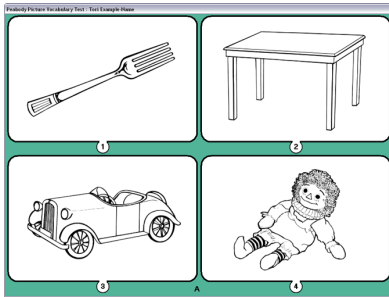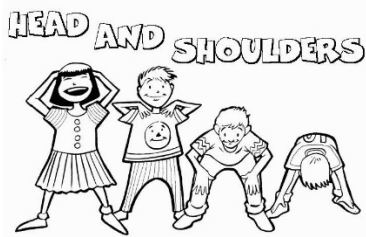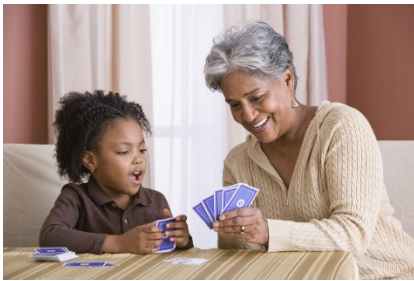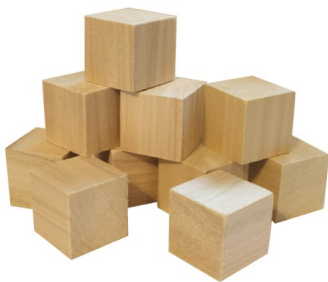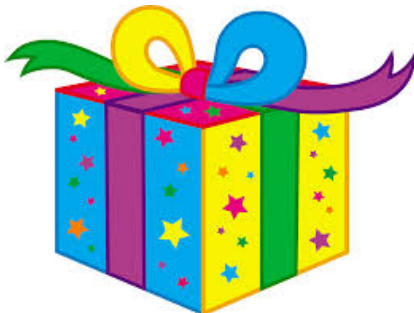

Supplement: Supplementary file 1 [file ijerph-17-05259-s001.zip › supplementary figure 1.pdf]
